# Assessment of the Periodontal Cementum Ablation Depth during Root Planing by an Er:YAG Laser at Different Energy Densities: An Ex Vivo Study

**DOI:** 10.3390/dj11050116

**Published:** 2023-04-27

**Authors:** Paul Nahas, Saad Houeis, Remi Chamboredon, Daniel Heysselaer, Toni Zeinoun, Samir Nammour

**Affiliations:** 1 Department of Restorative and Esthetic Dentistry, Faculty of Dental Medicine, Lebanese University, Hadath campus, Beirut 1003, Lebanon; paulnahas@ul.edu.lb; 2 Department of Dental Science, Faculty of Medicine, University of Liege, 4000 Liege, Belgium; saad.houeis@gmail.com (S.H.); remi.chamboredon@gmail.com (R.C.); daniel.heysselaer@gmail.com (D.H.); 3 Department of Oral and Maxillo-Facial Surgery, Dean of Faculty of Dental Medicine, Lebanese University, Beirut 1003, Lebanon; zeinountoni@gmail.com

**Keywords:** cementum, root planing, Er:YAG laser, periodontitis, laser, periodontal pocket

## Abstract

Introduction: An important and non-adapted delivered energy of Er:YAG laser can eliminate the total thickness of root cementum during root planing. Conversely, the preservation of a partial layer of cementum covering the roots is vital for any periodontal ligament regeneration. Thus, the assessment of the cementum ablation depth produced by each energy density of Er:YAG laser is essential before considering its use for the periodontal planing and treatment of the cementum and root surfaces. Aim of the study: Assessment of the cementum ablation depth at different energy densities of the Er:YAG laser is the aim of this study. Materials and methods: A total of 48 human caries free molars were collected and used in this study. Areas to be irradiated were delimited by two longitudinal grooves (0.5 mm depth). Roots were divided randomly into four groups (4 × *n* = 12). An Er:YAG laser (2.94 µm) was used with a side-firing tip (R600T) with a 600 µm diameter and a frequency of 20 Hz combined with a cooling system of air 6 mL/min and water 4 mL/min. We used a super short pulse mode (SSP: pulse duration: 50 μs). We used a single irradiation passage backward from apex to cervical parts at 1 mm/s with a slight contact and at an angle of 15° to 30° between the tip and the root surface. Different energies were selected: 30 mJ, 40 mJ, 50 mJ, and 60 mJ. Results: Microscopic observations showed that the average of the ablation depth increased with the increase of the delivered energy from 30 mJ to 60 mJ. Mean values of the ablation depths were respectively as follows: 43.75 ± 4.89 µm for the energy of 30 mJ, 50.05 ± 3.72 µm for 40 mJ, 65.56 ± 10.35 µm for 50 mJ, and 74.80 ± 15.23 µm for 60 mJ. A statistically significant difference existed between the ablation depth of all groups. Conclusion: Based on our results, the depth of cementum debridement is related to the level of the delivered energy. The lowest energy levels (30 mJ and 40 mJ) can ablate the root cementum surface for a variable depth from 43.75 ± 4.89 μm to 50.05 ± 3.72 μm.

## 1. Introduction

The major cause of periodontal inflammation is the bacterial colonization around the root surface. When biofilm and calculus build-up, they increase the risk of periodontal diseases [1]. Both bacterial biofilm and bacterial toxins colonize the periodontal hard tissue, leading to the deterioration of the cementum, as well as the loss of the periodontal attachment [2]. The most common treatment for this issue is the cementum Scaling and Root Planing (SRP). It is performed using adapted manual cleaning, sonic and ultrasonic devices, photodynamic therapy, and air-polishing.

In 1953, Glickman defined the importance of “scaling”, whereby calculus and biofilm had to be eliminated from supra and sub-gingival root’s surface, in order to treat periodontal diseases [3]. Later, in 1992, the process of “root planning” was introduced. The rough cementum, impregnated by toxins and bacteria, needed to be removed to transform the root’s surface into a smooth and clean surface [4,5]. Cementum apposition is a continuous process; throughout a tooth’s lifespan, its thickness can increase threefold with age. It varies from 16 µm to 60 µm in the coronal half. It may increase up to 200 µm in the apical region [6]. The maximal cementum thickness ranges between 5 and 800 µm in maxillary molars and between 20 and 700 µm in mandibular molars. In addition, more cementum apposition occurs lingually than labially in roots of mandibular molars as well as in buccal roots of maxillary molars. Moreover, the cementum accumulates in root concavities referred as anatomic depressions [7]. During root planing, total ablation of the cementum could cause dentin sensitivity, coupled with post-treatment pain [8]. It was suggested that one session of manual SRP should not remove more than 50 µm from the root’s surface and should preserve a part of the cementum in place. Furthermore, in order to eliminate toxins and bacteria and to achieve periodontal healing, it is not necessary to remove the complete thickness of the cementum [8]. Moreover, the preservation of a layer of the cementum is necessary to assure periodontal ligament regeneration and to initiate a periodontal ligament reattachment [9]. Unfortunately, surfacing deep periodontal pockets may not lead to a total cleaning and elimination of bacteria and their toxins [10]. Based on the study by Megally et al., when a pocket’s depth exceeds 5 mm, ultrasonic cleaning was only able to gain 1 mm of attachment and maintain the level of gum recession [11]. Additionally, in moderate periodontitis, non-surgical treatment can reduce the pocket’s depth and maintain the attachment level through decreasing gum inflammation [12].

The literature proposes several protocols to control periodontal diseases. In order to improve results of periodontal treatment, a variety of solutions were recently introduced, such as laser irradiation of gingival pockets [13]. Among different type of lasers, Erbium lasers such as neodymium-doped yttrium/aluminum/garnet (ND:YAG), erbium, chromium-doped yttrium/scandium/gallium/garnet (Er,Cr:YSGG), and erbium-doped yttrium/aluminum/garnet (Er:YAG), as well as high power diode lasers, are mainly used in SRP treatment [14]. Other type of lasers such as the CO_2_, Low Level Laser (LLL) have been used in photo-biomodulation therapy to decrease gingival inflammation and enhance the healing process by reducing the essential mediator in the acute inflammatory response known as prostaglandins [15,16]. Moreover, CO_2_ lasers have been reported to cause melting of root surfaces similar to Nd:YAG lasers [17,18]. Although the anti-bacterial and ablative effects of laser wavelengths have been discussed widely in the literature [19,20,21], limited laser wavelengths are able to conduct efficient cementum cleaning [22] and are able to create the lowest amount of surface roughness when low levels of energy have been used [23]. It is also important to mention that when Er:YAG lasers are used in SRP, neither cracks, microfractures, nor thermal side effects were observed on root surfaces [18].

Moreover, periodontitis treatment by lasers is still uncertain [24,25]. Therefore, the use of lasers in periodontal treatments needs to find a clear consensus regarding the type of lasers and the efficiency of energy density to be used. It was demonstrated that the Er:YAG laser wavelength is able to ablate dental hard tissue, selectively ablate calculus, and disinfect gingival pockets [26]. Laser periodontal treatment may be possible by the use of special tips, called “side-firing” tips, which can deliver laterally a laser beam. Lateral irradiation tips were presented in different designs; beveled at a specific degree ranging from 30 to 60 degrees for chisel tips, others can deliver a laser at 90 degrees. Lateral irradiation directs laser energy towards contaminated cementum. However, the important and non-adapted delivered energy of the Er:YAG laser can eliminate the total thickness of the root cementum. Conversely, the preservation of a partial layer of cementum covering the roots is vital for any periodontal ligament regeneration. Thus, the assessment of the cementum ablation depth produced by each energy density of Er:YAG laser is essential before considering its use for the periodontal planing and treatment of the cementum and root surfaces.

Therefore, the objective of this study is to assess the cementum ablation depth at different energy densities of Er:YAG laser, using side-firing tips. The null hypothesis supposes that there are no differences in ablation depth of the cementum, while using different energy densities.

## 2. Materials and Methods

### 2.1. Samples Preparation

**Figure 1:**
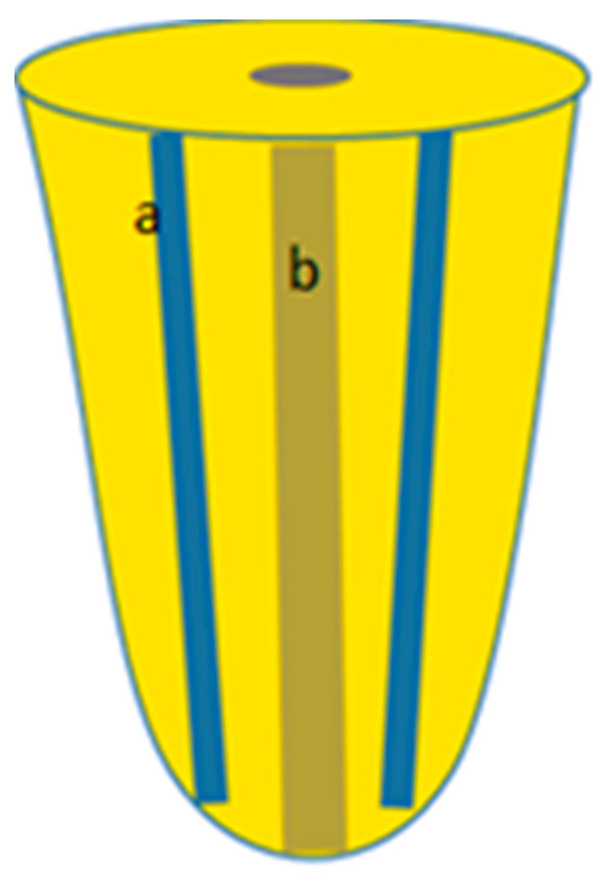
Root preparation. (**a**) longitudinal groove delimitation, (**b**) lased area.

A total of 48 caries-free human molars were collected. The extraction of teeth, for different reasons, is totally unknown by the authors and completely unrelated to our study. Wisdom teeth and molars with radicular decalcification, decay, or whatever pathology that could alter cementum layer were excluded. All teeth were slightly cleaned from all organic debris and tartar using an ultrasonic device and sectioned at the enamel–cementum junction. Then, roots were stored in distilled water until the beginning of the experiment. Two longitudinal grooves, at 0.5 mm depth, were performed with a diamond disk, along the roots, to delimit the irradiated area and to guide the microscopic observation (Figure 1). Roots were then divided randomly into 4 groups (*n* = 12).

### 2.2. Samples Irradiation

Only one operator conducted the whole experiment using Er:YAG (laser AdvErl Evo^®^, J. MORITA MFG. CORP., Kyoto, Japan), delivering a 2.94 μm wavelength equipped with a side-firing tip “R600T” (Tip “R600T”, J. MORITA MFG. CORP., Kyoto, Japan), with a 600 µm diameter. The frequency was adjusted to 20 Hz and combined with a cooling system of air 6 mL/min and water 4 mL/min. We used the super short pulse mode (SSP pulse duration: 50 μs). A single irradiation passage was performed on each root, starting from apical to coronal zone, in a backward motion, at an approximate inclination of the fiber to the root surface of 15° to 30° degrees and at a speed of 1 mm/s (operator dependent), in a mode of slight contact. Each group received different energy densities, respectively. G1 received 30 mJ; G2 received 40 mJ; G3 received 50 mJ; and G4 received 60 mJ. The energy densities per pulse related to each energy level were 10.7 J/cm^2^ for G1, 14.3 J/cm^2^ for G2, 17.8 J/cm^2^ for G3, and 21.4 J/cm^2^ for G4. Increased energy level was the only variable between groups affecting energy density values. All other parameters (pulse duration, Hz, beam diameter, and 1 mm/s irradiation velocity) were kept similarly in all our experiments.

### 2.3. Microscopic Observation

Non-decalcified samples were embedded into transparent resin (Epofix, Struers, Denmark), then cut transversally at a thickness of 100 μm using IsoMet 2000 (Buehler, Ltd., Lake Bluff, IL, USA), polished with 600 grit silicon papers (Matador, Germany), and then observed with a transmitted light microscope (AmScope ME580TA-PZ-2L-16M3, Irvine, CA, USA) at 40× magnifications. Samples were photographed with a digital camera with 16 megapixels (AmScope MU1603, Irvine, CA, USA). The depth of the ablated area between existing grooves was evaluated and calculated. To re-establish the original design of the root, a line was drawn starting from the highest points inside the longitudinal groove delimitation, using the 3-points circle measurement tool in AmScope software version x64, 4.11 (AmScope, Irvine, CA, USA). Two points of the mentioned tool were chosen on each side of the irradiated area, while the 3rd point was used to determine the curvature of the line. The ablation of cementum was measured using vertical line measurement tool within the same AmScope software from the newly drawn line to the deepest ablated area. When several depths appeared in the same crater (sample), multiple measurements were conducted. A mean value of the depth was then calculated per sample; then, mean and standard deviation were calculated per group.

### 2.4. Statistical Analysis

All statistical tests were done using GraphPad Prism 5 (GraphPad Software, Inc., San Diego, CA, USA). In order to compare the results of ablation depth of the cementum, a one-way ANOVA test was applied, coupled with the Newman–Keuls comparative test (post hoc test), to compare significant differences between all groups. The level of significance was set at *p* < 0.05.

## 3. Results

### 3.1. Optical Microscopic Observation

**Figure 2:**
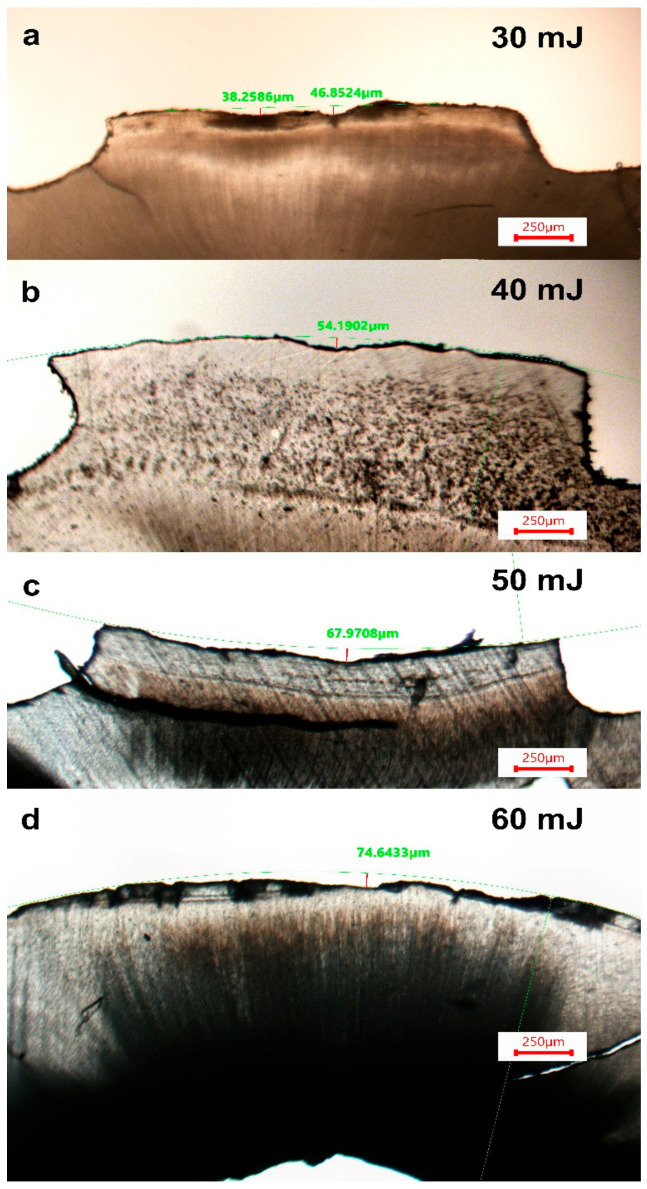
View of some samples showing different depths of cementum ablation caused by Er:YAG laser beam with different energies. (**a**) = 30 mJ, (**b**) = 40 mJ, (**c**) = 50 mJ and (**d**) = 60 mJ.

The cementum irradiation showed an irregular ablation depth and concave cavity bottom. Additionally, a systematic increase of the cementum ablation occurred as the energy of the Er:YAG laser increased (Figure 2). Results showed an increase in the mean depth of the ablated tissue with the increase of the energy from 30 mJ, to 60 mJ, respectively, as follows: 43.75 ± 4.89 μm for G1 (30 mJ), 50.05 ± 3.72 μm for G2 (40 mJ), 65.56 ± 10.35 μm for G3 (50 mJ), and 74.80 ± 15.23 μm for G4 (60 mJ) (Table 1).

### 3.2. Statistical Results

All results passed the Kolmogorov–Smirnov and Agostino and Pearson omnibus normality test (*p* < 0.05). One–way ANOVA showed a significant difference between groups with *p* < 0.0001. The Newman–Keuls post hoc test showed significant statistical differences between all groups: G1 and G2, G1 and G3, G1 and G4, G2 and G3, G2 and G4, and G3 and G4 with *p* < 0.05 (Table 1).

**Figure 3:**
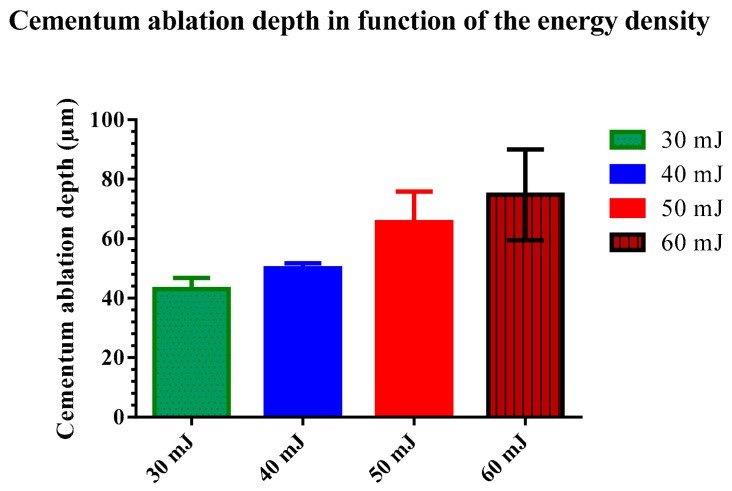
Bar graph showing the mean with standard deviation of the ablation depth in each group relating to the delivered energy.

The lowest cementum ablation depth appears at low energy irradiation of 30 mJ (Figure 3). The more the energy increases, the more the cementum ablation is visible. At the energy level of 60 mJ, we were able to see in few samples a total elimination of the cementum.

The null hypothesis is rejected for all energy levels (30 mJ, 40 mJ, 50 mJ, and 60 mJ) used for root irradiation with the Er:YAG laser.

## 4. Discussion

The use of side-firing Er:YAG laser irradiation for cementum ablation was investigated in this study. The efficiency of straight and beveled fiber optic tips in periodontal diseases was thoroughly studied in the literature [27,28,29]. In our study, the Er:YAG laser was used and, in combination with a side-firing tip, was able to ablate the superficial surface of the root’s cementum. The depth of cutting scored 43.75 ± 4.89 μm for the laser energy of 30 mJ and increased progressively until it reached 74.80 ± 15.23 μm with the increase of laser energy up to 60 mJ.

Human tooth root anatomy presents a lot of irregularities, curvatures, concavities, and furcation. Such specificities make ultrasonic and manual root planing difficult to totally clean the root surfaces. A study conducted by Caffesse et al. from 1986 on the efficiency of scaling and root planing (SRP) showed that 43% of root surfaces were free of calculus in pockets having 4 to 6 mm depth. Meanwhile, the percentage of a cleaned root’s surface, after SRP, dropped to only 32% with pockets deeper than 6 mm [30]. Additionally, Sherman et al. showed that 57% of root’s surface had residual calculus after ultrasonic and manual root planing, when observed under stereomicroscope [31]. Similar difficulties may occur during irradiation with a collimated light since laser tips can only be introduced in a gingival pocket parallel to the root direction. In our study, we had free access to the root surfaces, whereas, in narrow and deep clinical pockets, the tip (R600T) may have difficulty accessing and irradiating certain extremely narrow areas within the root anatomy, as well as difficulty reaching certain surfaces. On the other hand, the laser main energy density is delivered laterally, keeping the deepest sulcus attachment moderately impacted by laser irradiation. It was also mentioned by Gao et al. in 2021 that low energy levels delivered from the Er:YAG laser during root irradiation have no irreversible damage on periodontal tissues; on the contrary, it improves overall healing by reducing inflammatory factors and colony forming units [32]. Meanwhile, Yaneva et al. in 2016 demonstrated the innocuity of pulp tissue to the energy level (100 mJ, 50 Hz, and 40 s irradiation time) higher than the ones used in our study [33]. However, the literature is poor regarding the optimal energy to use with side-firing Er:YAG laser tips to fulfill the purpose of performing cementum cleaning and root planing while aiming to reduce the thickness of the ablated layer of this cementum and thus preserve as much as possible a residual layer of cementum which can allow the regeneration of the periodontal ligament. Moreover, there is a need to resolve the difficulties caused by different roots’ shapes and irregularities. When biofilm and bacterial toxins infiltrate the external surface of the root’s cementum and the epithelial sulcus walls and when patient hygiene is insufficient to control gingival or periodontal inflammation, deep pockets must undergo surfacing and minimum cementum ablation [34].

Preserving a layer of cementum is essential since it contains a unique molecule, the so-called “cementum attachment protein”, which supports the adhesion of gingival fibroblasts and keratinocytes that are essential for any ligament regeneration and against bacterial infiltration [35]. Mlachkova et al. showed that pocket’s depth decreases after manual debridement [12]. To improve periodontal healing, different types of lasers have been analyzed by the American Academy of periodontology; laser-assisted periodontal therapy could enhance the reduction of pocket’s depth, as well as post-operative bleeding, especially with pockets deeper than 5 mm and in patients under anti-coagulants following surgical treatment [36]. The Er:YAG laser results could be related to the mode of firing of the laser beam and to the content of water in cementum, evaluated to be 22% by weight, considering that the Er:YAG laser absorption length in water is around 0.7 µm [20,36] and has a coefficient of absorption of 10^4^ cm^−1^ [37]. Such strong absorption of ER:YAG laser light is responsible for the sudden heating of water and micro-explosions inside the dental hard tissue, causing cementum removal with minimal damage as a result of the cooling air/water spray system during irradiation [37,38]. Moreover, Sasaki et al. showed a significant difference in tissue composition, between organic and inorganic components, after Er:YAG irradiation, where the loss of proteins components (more hydrated) is higher than that of hydroxyapatite, which may explain, by extrapolation, the effect of the laser on highly hydrated bacteria present in periodontal inflammation [37]. In vivo, bacterial load into pockets decreased with the use of the Er,Cr:YSGG laser at 40 mJ. This decrease happens until the 6-month point of irradiation of periodontal pockets with an RFPT 5-14 tip, which irradiates the sulcus in a 360-degree conical shape, where 80% of the energy density goes laterally, and only 20% goes straight from the tip toward the attached ligament [27]. Additionally, decreases in bacteria inside pockets have been demonstrated after using the Er:YAG laser at 160 mJ [39]. Moreover, the Er:YAG laser at 100 mJ and 15 Hz was able to reduce lipopolysaccharides (LPS), known as inflammatory factors in periodontitis, by 83.1%, with less working time than an ultrasonic procedure [40]. Furthermore, the level of endotoxins observed in periodontitis decreased significantly when Er:YAG and Nd:YAG lasers were used in combination with normal scaling [19]. The same conclusion was presented by Soundarajan et al. in 2022, where adjunctive SRP with an Er,Cr:YSGG laser, operating at 1 W with water calibrated to 15% and air calibrated to 10%, gave better clinical results after 3 months of follow-up than SRP alone [41]. According to Folwaczny et al., after Er:YAG laser irradiation (Key II; Kavo; Biberach; Germany; pulse repetition rate: 15 pps; pulse duration: 250 µsec with glass chisel tip), the endotoxin LPS average on the root surfaces was significantly decreased by 19.86 (±14.4) IU/mL at 60 mJ while the mean of the LPS from the untreated control samples was 50.1 ± 35.9 IU/mL [42]. On the contrary, the use of a diode laser at 980 nm did not improve either the overall periodontal clinical parameters or the bacterial load and did not reduce the pocket biofilm when compared to standard scaling (SRP) [29]. Additionally, the CO_2_ laser showed an important alteration of root surface when operated at 2 W with an energy density of 2.7 J/cm^2^ and a frequency of 2 Hz. On the other hand, the same parameters were able to stimulate periodontal cell and induce re-attachment [43]. Thus, not all wavelengths seem to improve periodontal pocket health the same way.

On the other hand, to improve periodontal health, it is essential to eliminate tarter, bacteria, endotoxins, and the infiltrated cementum surface, either manually or combined with laser. In our study, we tried to assess the efficiency of Er:YAG photomechanical ablation, at low level of energy starting at 30 mJ and going up to 60 mJ, by calculating the mean cementum ablation depth, which ranges from 43.75 µm to 74.80 µm, respectively. Frenzten et al. have shown a higher mean of cementum ablation depth (77.1 µm), when the Er:YAG energy level increased to 100 mJ, with a 1.10 tip emitting laser, within an arc of +30 degrees to −30 degrees [44]. Increasing laser energy level may not be needed, since debridement causes less crater depth in the range of 15.6 µm for manual and 5.3 µm for ultrasonic instrumentation [44]. Furthermore, Bozbay et al. showed that the mean loss of cementum in manual instrumentation ranges from 22.28 µm to 23.67 µm and from 13.08 µm to 14.50 µm for piezoelectric instrumentation [45]. For that reason, we decided, in our study, to select and use the lowest levels of energy, ranging from 30 mJ to 60 mJ. Unfortunately, the actual available laser apparatus equipped with a side-firing tip cannot yet deliver lower energies below 30 mJ. Despite the thickness of the ablated cementum, it is known that the infiltrated cementum surfaces should be eliminated to decrease the bacterial contamination effect and to initiate the healing process of the surrounding tissues.

Scaling and root planing (SRP) produce slight ablation of the contaminated cementum surfaces, resulting in a new residual cementum surface. This remaining cleaned cementum surface appears to be more biocompatible for periodontal healing and specifically for tissue regeneration. The choice of Er:YAG laser wavelength has the advantage of partial and superficial removal of the cementum without creating surface cracks [44], which help keeping initial root surface hardness and specificity. Moreover, the cementum surface had a better resistance and was not affected even by minor decalcification after Er:YAG laser debridement at 30 mJ/pulse, 30 Hz, and a pulse duration of 200 µs, with a straight tip of 600 µm angled at 30 degrees [46]. None of our observed samples showed any image of cracks in all groups and at all levels of energy. This confirms that the energy levels chosen in our experiment fall within a safe range for cementum ablation. Moreover, the lowest energy used (30 mJ) was capable of cutting, from cementum, enough thickness, even more than of conventional technics for cementum debridement. Additionally, the higher energy levels showed a significantly higher ablation depth, presenting a higher risk of complete root cementum removal. With the Er:YAG laser, the lower the energy, the less ablation, which produces a lower risk of total removal of the cementum layer. With the Er:YAG laser, the shorter the pulse duration, the more the ablation is efficient, is deeper, and generates minimal temperature increase [20]. Therefore, we have chosen in our experimental model the SSP (Super Short pulse) mode. Moreover, the lowest energy density used (30 mJ), in combination with a very short pulse duration and side-firing tip, resulted in a cementum ablation depth around 40 µm, with only one passage of irradiation. Such fast debridement, as well as their results, is similar to the other published studies. Sasaki et al. used a sweeping motion along the root for 20 s [37]; Maruyama et al. used a sweeping motion of 45 s [46]; Almehdi et al. used a sweeping motion in a line of irradiation for 2 s [47]. In our study, we treated root surfaces at a speed of 1 mm/s. Thus, the total irradiation time is dependent on the root length. For a root of 10 mm length, our total irradiation time was around 10 s.

Further studies are needed to confirm our obtained results from Er:YAG laser irradiation, regarding cementum ablation depth, especially for lower energy levels that may minimize more the ablation depth of the dental root cementum. This depth reduction may allow more preservation of the precious residual cementum layer essential for any future periodontal ligament regeneration. Moreover, long term clinical studies followed by periodontal changes assessment are important since our study is limited to ex vivo evaluation without the presence of any biological interaction.

## 5. Conclusions

Based on our results, the depth of cementum debridement is related to the level of the delivered energy. The lowest energy levels (30 mJ and 40 mJ) of the Er:YAG laser used with a side-firing tip R600T can ablate the root cementum surface for a variable depth from 43.75 ± 4.89 μm to 50.05 ± 3.72 μm. Higher energy levels can create deeper crater in cementum up to 65.56 ± 10.35 mm and 74.80 ± 15.23 mm for the energy levels of 50 mJ and 60 mJ, respectively.

## Figures and Tables

**Table 1:** Mean depth of ablated cementum in μm with standard deviation for each group.

Group	G1	G2	G3	G4
Energy	30 mJ	40 mJ	50 mJ	60 mJ
Mean	43.75 ^A^	50.05 ^B^	65.56 ^C^	74.80 ^D^
Std. Deviation	4.89	3.72	10.35	15.23

Similar superscript letters mean no significant differences between groups, while different letters attest a significant difference between groups.

## Data Availability

Not applicable.
